# Testing implementation facilitation of a primary care-based collaborative care clinical program using a hybrid type III interrupted time series design: a study protocol

**DOI:** 10.1186/s13012-018-0838-2

**Published:** 2018-11-29

**Authors:** Amanda M. Midboe, Steve Martino, Sarah L. Krein, Joseph W. Frank, Jacob T. Painter, Michael Chandler, Allison Schroeder, Brenda T. Fenton, Lara Troszak, Taryn Erhardt, Robert D. Kerns, William C. Becker

**Affiliations:** 1 Center for Innovation to Implementation (Ci2i), VA Palo Alto Health Care System, Stanford University, 795 Willow Rd (152-MPD), Menlo Park, CA 94025 USA; 2 Pain Research, Informatics, Multimorbidities and Education (PRIME) Center, VA Connecticut Healthcare System, West Haven, CT USA; 3 Yale School of Medicine, New Haven, CT USA; 4 Center for Clinical Management Research (CCMR), VA Ann Arbor, Ann Arbor, MI USA; 5 University of Michigan School of Medicine, Ann Arbor, MI USA; 6 VA Eastern Colorado Healthcare System, HSR&D Center for Veteran-Centered and Value-Driven Care, Denver, CO USA; 7 University of Colorado School of Medicine, Aurora, CO USA; 8 Center for Mental Healthcare and Outcomes Research, Central Arkansas Veterans Healthcare System, North Little Rock, AR USA; 9 Division of Pharmaceutical Evaluation and Policy, University of Arkansas for Medical Sciences, Little Rock, AR USA

**Keywords:** Opioids, Primary care, Collaborative care, Benzodiazepines, Implementation, Hybrid design, Pharmacist

## Abstract

**Background:**

Dissemination of evidence-based practices that can reduce morbidity and mortality is important to combat the growing opioid overdose crisis in the USA. Research and expert consensus support reducing high-dose opioid therapy, avoiding risky opioid-benzodiazepine combination therapy, and promoting multi-modal, collaborative models of pain care. Collaborative care interventions that support primary care providers have been effective in medication tapering. We developed a patient-centered Primary Care-Integrated Pain Support (PIPS) collaborative care clinical program based on effective components of previous collaborative care interventions. Implementation facilitation, a multi-faceted and dynamic strategy involving the provision of interactive problem-solving and support during implementation of a new program, is used to support key organizational staff throughout PIPS implementation. The primary aim of this study is to evaluate the effectiveness of the implementation facilitation strategy for implementing and sustaining PIPS in the Veterans Health Administration (VHA). The secondary aim is to examine the effect of the program on key patient-level clinical outcomes—transitioning to safer regimens and enhancing access to complementary and integrative health treatments. The tertiary aim is to determine the categorical costs and ultimate budget impact of PIPS implementation.

**Methods:**

This multi-site study employs an interrupted time series, hybrid type III design to evaluate the effectiveness of implementation facilitation for a collaborative care clinical program—PIPS—in primary care clinics in three geographically diverse VHA health care systems (sites). Participants include pharmacists and allied staff involved in the delivery of clinical pain management services as well as patients. Eligible patients are prescribed either an outpatient opioid prescription greater than or equal to 90 mg morphine equivalent daily dose or a combination opioid-benzodiazepine regimen. They must also have an upcoming appointment in primary care. The Consolidated Framework for Implementation Research will guide the mixed methods work across the formative evaluation phases and informs the selection of activities included in implementation facilitation. The RE-AIM framework will be used to assess Reach, Effectiveness, Adoption, Implementation, and Maintenance of PIPS.

**Discussion:**

This implementation study will provide important insight into the effectiveness of implementation facilitation to enhance uptake of a collaborative care program in primary care, which targets unsafe opioid prescribing practices.

**Electronic supplementary material:**

The online version of this article (10.1186/s13012-018-0838-2) contains supplementary material, which is available to authorized users.

Evidence supporting the use of opioids for chronic pain is modest [1, 2], and serious safety, misuse, and abuse concerns have increased over the last two decades [3–7]. In 2017, the opioid overdose epidemic was declared a public health emergency, and the Council of Economic Advisers estimated the cost to the USA was $504 billion in 2015 alone [8]. To decrease morbidity and mortality in the context of therapeutic use of opioid therapy for the management of pain, a growing body of research and expert consensus support reducing high-dose opioid therapy, avoiding opioid-benzodiazepine combination therapy, and promoting multi-modal pain care in which evidence-based complementary and integrative health (CIH) treatments are incorporated alongside pharmacologic treatment [9, 10].

The Veterans Health Administration (VHA) launched the Opioid Safety Initiative (OSI) in early 2013 as part of a larger strategy to respond to the opioid epidemic. The OSI’s objectives include engaging patients in discussions about safer medication treatment regimens. While there has been some success in decreasing the number of patients prescribed risky opioid regimens [11], meeting the challenge of de-implementing high-risk medication regimens using patient-centered methods is still a major concern across the VHA [12].

To address the needs of clinicians at VHA facilities, we designed a collaborative care clinical program, known as Primary Care-Integrated Pain Support (PIPS). The intent of PIPS is to decrease the proportion of patients on high-risk opioid regimens for chronic pain and increase the proportion of patients treated with CIH treatments, all while preserving or improving quality of life and functional status. PIPS was based on a successful model of a pharmacist-led collaborative care intervention in VHA [13], qualitative work that identified the need for improved coordination of pain care and communication among providers [14], and review of similar evidence-based interventions that aimed to decrease benzodiazepine use [15] or decrease opioid use [16]. Specific components of the intervention include letters inviting patients to ask providers about PIPS, primary care provider education about PIPS, a PIPS referral template for primary care providers, and a structured program in which clinical pharmacists work one-on-one with patients to transition them to safer regimens and increase access to CIH treatments. Clinical pharmacists are key PIPS providers, working closely with primary care teams to taper medications and motivate and refer patients to CIH treatments.

Collaborative care models, where non-physicians provide support and care coordination to high-need patients, have consistently shown benefit in chronic pain studies, particularly in the VHA [17–19]. However, these models are not typically well-integrated into regular clinical practice without concerted implementation support. Implementation facilitation has been used successfully to increase the uptake of evidence-based practices and programs (EBPPs) in primary care and other clinical settings [20]. It is a multi-faceted strategy that focuses on supporting individuals and groups in adapting EBPPs for their setting and problem-solving around implementation barriers. As part of implementation facilitation, external and internal facilitators work together to support implementation through various discrete implementation activities [21]. The remainder of this section will focus on describing the details of the PIPS clinical practice as well as the implementation strategy, followed by a description of the aims of this study.

## Clinical practice

Patients eligible for PIPS have (1) an upcoming primary care appointment within 2–4 weeks at one of the three sites and (2) active opioid prescriptions at any VHA facility totaling 90 mg morphine equivalent daily dose (MEDD) or more *or* active, concurrent opioid and benzodiazepine prescriptions. Initiation of PIPS engagement with these patients is as follows:

1. Local clinic staff mail a letter to eligible patients 2–4 weeks in advance of a routine primary care appointment. The letter explains that there are safety concerns around opioid prescriptions and that PIPS may help the patient reduce medication use and engage in CIH treatments. Patients are encouraged to bring the letter with them to their upcoming primary care appointment to discuss with their providers.
2. During the appointment, the primary care provider discusses medication safety concerns and the rationale for CIH treatments and if the patient agrees, refers them to PIPS via a templated pharmacy PIPS consult. Providers may also refer eligible patients who have not received a letter (e.g., incorrect mailing address).
3. For PIPS-referred patients, a medical assistant schedules an intake appointment for the patient with the clinical pharmacist. The objectives of the first appointment are to build rapport, review the patient’s preferences for CIH treatment, initiate the taper plan if the patient agrees, and set a schedule for follow-up. The clinical pharmacist uses a guideline for dose tapering that specifies dose and number of pills supplied at each 2-week interval but can vary from the guideline as dictated by clinical circumstances. If a dose reduction is planned, but the patient is unwilling, the clinical pharmacist uses clinical judgment to alter the schedule for PIPS follow-up, as needed.
4. The clinical pharmacist follows up with the patient based on patient needs. Follow-up sessions may be conducted by telephone or in-person depending on the patient’s preference and clinical course. The topics covered include progress with medication changes, quality of life and pain-related functional interference, patient safety, and engagement in CIH treatments. In addition to overseeing patients’ tapering progress, the clinical pharmacist places referrals for patients’ preferred CIH treatments, tracks patients’ attendance, and addresses any barriers to CIH treatment engagement. Primary care providers are tagged to clinical pharmacist notes, so they are made aware of the progress of tapers and expected date of patients’ discharge from PIPS. A standardized note template is used at each site to consistently track patient progress over time. Patients may stay enrolled in PIPS for up to 6 months based on their needs.

## Implementation strategy

We will use an implementation facilitation strategy [20] to enhance uptake and sustainability of PIPS. An external facilitation (EF) team, consisting of clinical and implementation experts, will work with an internal facilitation (IF) team at each site, consisting of an internal facilitator and local champion. The EF team includes a physician and two clinical psychologists with expertise in implementation. The physician is an expert in PIPS delivery and will serve as the primary external facilitator, whereas the two psychologists will consult with the EF and IF team as needed. The internal facilitator within the IF team is an experienced physician familiar with the facility’s procedures, organizational structures, clinical processes, and culture. The local champion within the IF team is a clinical pharmacist who provides formal marketing, including emails or presentations and directly interfaces with primary care physicians. The EF and the IF team will meet at least twice per month by phone and communicate as needed in between regular meetings.

In addition, we will use a case-finding dashboard [22] to identify PIPS-eligible patients across the three sites. Local site staff will use the dashboard bi-weekly to view lists of patients and generate letters for mailing to patients with upcoming primary care appointments. Table 1 provides a description of each of the implementation facilitation activities being applied to PIPS implementation; these activities were drawn from existing literature [22].

**Table 1:** Implementation facilitation activities

Activity	Definition
Engagement with the facilitation team	The IF and EF teams engage during monthly project-wide and separate site-specific team calls. The IF team also contacts the EF team whenever questions arise related to PIPS implementation, while the EF team engages the IF team outside of scheduled calls when the need arises.
Academic detailing/education	Educating providers and key stakeholders about PIPS or other pain management assessment and treatment tools. This strategy involves direct outreach by the IF team and sometimes the EF team to educate providers at a given site. The intent is to promote providers’ use of PIPS through clear and regular education about the program and pain management strategies, including CIH treatments. This strategy can include one-on-one interactions with providers about PIPS treatment, PIPS educational meetings with different stakeholder groups (e.g., providers, administrator, veteran patient/consumer group, family stakeholders), assessment of PIPS knowledge and motivation to enroll patients in PIPS, and providing positive feedback for improved clinical practices as they relate to PIPS objectives.
Problem-solving	This activity is based on ongoing assessment, either formal (interviews, surveys) or informal (conversations), of implementation barriers to PIPS implementation. This information is used to troubleshoot challenges as they arise, using regularly scheduled site-specific and project-wide calls or communication in between meetings as necessary.
Audit and feedback	This activity is conducted monthly unless another schedule is deemed necessary. In brief, it involves collecting and summarizing clinical performance data related to PIPS (e.g., number referred, enrolled, completed) and providing that feedback to the IF and local champion at the site. This activity allows the implementation team to review progress and adjust behavior as necessary to enhance enrollment.
Capturing and sharing local knowledge	This activity occurs on the project-wide calls, but may also occur through informal communication (e.g., email). This activity is meant to help sites capture and share what is working to facilitate PIPS implementation in their clinical setting.
Changing record systems	Altering record systems as needed for PIPS implementation. The main records for PIPS are the letter to patients (explaining PIPS), a consult template for referral to PIPS, and intake and progress note templates stored in the electronic medical record. Each site can modify these records to fit their culture and care processes.
Marketing and distribution of materials	This activity typically involves the use of flyers and announcements in meetings or via email. It may occasionally overlap with academic detailing in that some marketing materials will be educational, describing key PIPS components and potential benefits to patients and providers.
Changing the clinical teams	This activity involves altering the team that provides care to PIPS patients, whether that be through replacing providers, adding new members, or changing duties and responsibilities. For example, if a new clinical pharmacist is brought into the team to help with referrals to PIPS, then that would represent a change in the clinical team.
Changing the implementation team	Changing or adding an IF, EF, or local champion to the implementation team.
Developing materials and adding them to a shared library	Placing PIPS-related materials in a shared library (computer-based or other) for the purposes of making it easier to access items and information that could be used to teach clinical team members or other stakeholders about PIPS and for providers to deliver the program.
Informing local opinion leaders	Members of the implementation team reach out to providers or administrators at a site who are educationally influential to inform them about PIPS and encourage them to support PIPS implementation (e.g., by mentioning PIPS at a staff meeting or talking about the benefits of PIPS for their patients).
Engaging national and regional policy makers	This activity entails the EF or IF teams reaching out to national or regional leadership about PIPS, informing them about implementation progress, or soliciting feedback on potential modifications.
Providing technical assistance and coaching	This activity is intended to help the IF teams and local staff deliver a high-quality PIPS clinical program. It can include providing assistance on the use of the automated case-finding dashboard tool or consult template or helping the provider improve their PIPS-related skills (e.g., adjusting medication tapering protocols, providing recommendations for CIH treatments).
Participation in a community of practice (COP)	The COP meeting occurs monthly for IF team members and providers and is led by the clinical expert of the EF team. Given the challenges and skills required to work with patients on opioid tapering and motivating them to engage with CIH treatments, the purpose of the COP is to advance accurate knowledge, improved skills, and positive attitudes when working with patients in the PIPS program.

## Aims

The overarching objective of this study, following an implementation-effectiveness hybrid type III design [23], is to understand how to facilitate uptake of the PIPS clinical practice. The primary aim is to determine if implementation facilitation leads to uptake and sustained use of PIPS and which activities within implementation facilitation are the active components leading to successful implementation. The secondary aim is to examine the effectiveness of PIPS on clinical outcomes, including transitions to safer medication regimens and uptake of CIH treatments. The tertiary aim will determine the budget impact of implementation of PIPS, which will provide VHA leadership with a cost estimate to inform decisions about future spread of PIPS [24]. The three major time periods or phases are (1) an 18-month pre-implementation period, (2) an 18-month implementation period, and (3) a 6-month sustainment period.

## Methods

In this section, the study sites will be described, followed by the evaluation framework, study participants, measures, data collection, and analysis plans for each study aim. The study protocol was approved by the institutional review boards at the site of the principal investigator (VA Connecticut Healthcare System) and at the PIPS implementation sites. A waiver of informed consent for patients and providers was approved, as the PIPS clinical program was deemed not human subjects research.

### Study sites

The PIPS clinical program and accompanying implementation facilitation strategy is being employed and evaluated at three VHA health care systems: VA Eastern Colorado Health Care System (ECHCS), Central Arkansas Veterans Healthcare System (CAVHS), and Tennessee Valley Healthcare System (TVHS). At the VA ECHCS, the main facility and two affiliated outpatient clinics are included in PIPS implementation. At the CAVHS, the two main facilities and eight affiliated outpatient clinics are included in PIPS implementation. At the TVHS, the two main facilities and two affiliated outpatient clinics are included in PIPS implementation. Multiple primary care clinics are located within the main facilities and outpatient clinics. The sites all have strong clinical expertise in opioid safety but vary in the degree to which multi-modal pain care and opioid safety initiatives have penetrated primary care practice.

### Aim 1: Implementation evaluation

Implementation will be assessed in two ways: (1) mixed methods formative evaluation during the pre-implementation, implementation, and sustainment phases to monitor our approach to implementation facilitation and progress [25] and (2) fidelity to implementation facilitation activities.

Each phase of formative evaluation has different objectives. During the 18-month pre-implementation phase, we will identify barriers and facilitators prior to implementation. During the 18-month implementation phase, implementation progress will be monitored and implementation activities modified as needed. During the 6-month sustainment phase, we will gather data related to successes or failures after implementation, including stakeholders’ perceptions of the utility of PIPS, satisfaction with the implementation process, and recommendation for future implementation refinements. The implementation facilitation fidelity assessment will allow us to determine the degree to which certain implementation facilitation activities (Table 1) were used during the implementation period and how they relate to the uptake of PIPS.

#### Evaluation framework

The study relies on two complementary implementation frameworks—the RE-AIM framework [26, 27] and the Consolidated Framework for Implementation Research (CFIR) [28]. Four dimensions within RE-AIM will be used to assess implementation—Reach, Adoption, Implementation, and Maintenance (with the Effectiveness domain being applicable to the secondary aim of this study).

*Reach* refers to the proportion of patients touched by PIPS. *Adoption* refers to the proportion of providers who use PIPS. *Implementation* refers to adaptations made to improve uptake of the clinical practice as well as fidelity to the PIPS protocol. *Maintenance* refers to whether PIPS is maintained over time and will be assessed during the 6-month, sustainment period.

The CFIR is a meta-theoretical framework that includes five domains and 39 constructs that influence the implementation of EBPPs. The CFIR was selected to guide the mixed methods formative evaluation to identify facilitators and barriers to implementation as well as the chosen implementation facilitation activities. Since not all 39 constructs were relevant to the clinical practice we are implementing, we selected the CFIR constructs per domain most relevant to PIPS implementation and tied them to the previously outlined specific implementation facilitation activities (see Table 2).

**Table 2:** CFIR domains, constructs, and implementation facilitation activities

CFIR domain	CFIR constructs	Implementation facilitation activities
Intervention characteristics	-Evidence strength and quality	-Engagement with facilitation team
	-Relative advantage	-Academic detailing
	-Adaptability	-Audit and feedback
	-Design quality and packaging	-Change clinical team
	-Cost	-Develop and share materials
		-Inform local opinion leaders
Inner setting	-Networks and communication	-Academic detailing
	-Culture	-Problem-solving
	-Implementation climate	-Audit and feedback
	-Tension for change	-Capture and share local knowledge
	-Compatibility	-Change record systems
		-Marketing and distribution of materials
		-Change implementation team
		-Provide technical assistance and coaching
Outer setting	-Patient needs and resources	-Engagement with facilitation team
	-External policy and incentives	-Engaging national and regional policy makers
		-Community of practice
Characteristics of individuals	-Knowledge and beliefs about the intervention	-Engagement with facilitation team
		-Academic detailing
		-Develop and share materials
		-Inform local opinion leaders
Process	-Engaging	-Academic detailing
		-Marketing and distribution of materials
		-Inform local opinion leaders

#### Mapping of CFIR to implementation facilitation activities

The intervention characteristics domain refers to attributes of interventions that influence successful implementation. Within this domain, there are five key constructs that could affect PIPS implementation, ranging from evidence strength and quality to cost. Facilitation activities that can be used to address these issues include engagement with the facilitation team and academic detailing.

The inner setting domain refers to features within the local context that can influence the PIPS implementation. Three key constructs—networks and communication, culture, and implementation climate—are important matters for PIPS implementation. For example, to enhance compatibility, we will capture and share local knowledge among leadership and providers and engage in problem-solving as issues arise.

The impact of the outer setting domain may be mediated through the inner setting, but certain economic, social, and political characteristics may impact implementation success. For this implementation work, we are interested in two key constructs: patient needs/resources and external policy/incentives. For example, to target patient needs and resources, we use a community of practice to troubleshoot potential barriers to patients receiving care.

The role of individuals in implementation success cannot be underestimated. For PIPS implementation, individuals’ knowledge and beliefs about interventions is most relevant. To address this construct, providers receive academic detailing and materials are developed for sharing among key staff.

Within the process domain, we are primarily interested in engaging key opinion leaders and making sure all appropriate personnel receive education and training related to PIPS.

### Study participants

Participants include stakeholders at each facility engaged in pain management, including clinicians (e.g., pharmacists, primary care providers) and clinic and facility-level leadership.

### Data collection

#### Administrative data

Data for the *Reach*, *Adoption*, and *Maintenance* dimensions will be obtained from VHA administrative data pulled directly from the electronic medical record (i.e., consults, clinical notes for enrolled PIPS patients) across the relevant implementation or sustainment periods.

#### Staff interviews

The CFIR informs the development of all semi-structured interview guides. Interviews will be over the phone but may occur in person during a site visit and are used across all phases of formative evaluation (pre-implementation, implementation, and sustainment phases) to obtain information about factors that might support or hinder PIPS implementation and to inform the use of implementation facilitation. Interviews will be audio-recorded and transcribed verbatim using VA’s centralized transcription service program.

#### Organizational change measure

We will assess organizational readiness using the Organizational Readiness for Implementing Change (ORIC) instrument [29]. The ORIC is a reliable and valid ten-item survey measure for assessing the shared belief among members of an organization that their organization is ready for change. It has two subscales: change commitment (resolve to implement a change) and change efficacy (collective capability to implement a change). Each item is rated using a five-point ordinal scale, ranging from “disagree” to “agree.” The total score ranges from 10 to 50, with higher scores indicative of greater organizational readiness for change.

#### Implementation facilitation training and fidelity

Two implementation facilitation experts (clinical psychologists with implementation facilitation training) will provide training to the EF and the IF teams to enhance fidelity to implementation and ensure implementation facilitation activities are recorded for fidelity monitoring. The training will include (1) descriptions of the implementation facilitation activities, (2) examples of these activities in the context of PIPS, and (3) how to record these activities in a monthly log. The EF and IF team members will receive either group or one-on-one training on a call by at least one of the implementation facilitation experts prior to the start of implementation. Training will be delivered in person whenever possible, but may also be delivered by phone when an in-person visit is not feasible. The training materials include a 60-min slideshow presentation and three sample vignettes that illustrate implementation facilitation activities (see Additional file 1). The presentation describes each of the implementation facilitation activities and provides examples relevant to PIPS implementation. The sample vignettes describe fictitious PIPS implementation efforts intended to promote understanding and accuracy when identifying and recording implementation activities. The expert trainer(s) will review each vignette one at a time and ask trainees to identify which implementation facilitation activities are occurring in the given vignette, addressing any questions when they arise. For the last portion of the training, the trainees will be instructed on how to complete a monthly online implementation facilitation strategy log for recording all facilitation activities. After the training, each IF team member will be asked to independently review eight additional vignettes for the presence or absence of implementation facilitation activities and return them to the trainers within 1 week of training. The expert trainers will review each of the trainee’s responses and provide feedback or additional training if warranted.

During the PIPS implementation phase, the EF and IF team members will complete a 10- to 15-min facilitation log each month, distributed via REDCap (a secure web application). The items include questions about the frequency of engagement in, and duration of, the various implementation facilitation activities and free-text fields for elaboration on these activities (see Additional file 2). The monthly assessment will allow us to directly measure how certain facilitation activities influence PIPS implementation over time.

### Data analysis

*Reach* is determined by tracking the number and proportion of eligible patients at the site during implementation who enrolled in PIPS monthly (i.e., number of patients enrolled in PIPS divided by eligible patients). *Adoption* by a provider is defined as the referral of one or more patients to PIPS. To calculate provider adoption, the case-finding dashboard archives all primary care providers who have an eligible patient at the site during the implementation period. We will then divide the number of providers who made at least one referral by the total number of providers, again relying on VHA administrative data. Measuring *Maintenance* involves tracking the proportion of eligible patients referred to PIPS monthly, comparing implementation and sustainment time-periods using VHA administrative data.

#### Staff interviews

Grounded theory methodology will be used for the qualitative analysis of interview transcripts [30, 31]. Specifically, open coding will be conducted initially, identifying key concepts that emerge from the participants’ language. Descriptive phrases (codes) will be assigned to segments of text, relying on qualitative analysis software to facilitate coding and sorting of data. Similarities and differences in themes will be examined by coders as part of a constant comparisons analysis. These themes will be discussed with the research team and refined through discussion.

#### Fidelity

The ability of the raters to correctly identify the absence/presence of specific implementation facilitation activities is tested using seven vignettes. Following each vignette, raters will be asked to identify which, if any, of fourteen implementation facilitation activities are utilized. Ratings will be calculated for the appraisal of each implementation facilitation activity across the vignettes. The established gold standard is the study leadership’s inclusion/exclusion of an implementation facilitation activity in a given vignette. The denominator used for each calculation will be based upon the number of raters by the number of vignettes total (total = 80). Sensitivity and specificity will be calculated to determine participants’ ability to accurately identify the activities described in the vignettes. For scores not in acceptable ranges (< .75), additional training will be conducted.

### Aim 2: Effectiveness evaluation

*Effectiveness* refers to the degree to which PIPS meets clinical goals. The clinical goals of PIPS are to transition patients to safer medication regimens and to expand uptake of CIH treatments. We define the transition to a safer medication regimen as the reduction of opioid dose from greater than or equal to 90 mg MEDD to less than 90 mg MEDD, or as the transition from combination opioid-benzodiazepine receipt to only one or the other (i.e., discontinuation of one medication class) without a dose increase in the other. The uptake of a CIH treatment is defined as receipt of a CIH treatment appointment. CIH treatments include rehabilitation medicine as well as complementary and integrative medicine treatments (e.g., acupuncture).

#### Study participants

Patient eligibility criteria for a monthly cohort are (1) having an active opioid prescription greater than or equal to 90 mg MEDD for the treatment of chronic non-cancer pain OR concurrent opioid and benzodiazepine prescriptions (regardless of dose) and (2) having a routine primary care provider visit coming up within a month.

#### Data collection

Data will be obtained from the VHA’s administrative data, relying on outpatient visit and prescription data sources. Identification of CIH treatments will be based on previous administrative data-based work [32].

#### Data analysis

We will assess the effectiveness of PIPS in safely achieving its clinical targets using an interrupted time series (ITS) design with three study periods (18 months of pre-facilitation, with 18 months of facilitation, and 6 months of sustainment) divided into monthly time cohorts. Veterans will be assigned to the time cohort representing the month in which they met eligibility criteria for PIPS and will be followed prospectively to observe whether they transition to safer medication regimens or utilize CIH treatments. Monthly rates will be assessed, using mixed effects logistic regression models that will incorporate indicators for the study period, variables capturing time elapsed since the beginning of each study period, site random effects, and patient characteristics. Control sites will be selected for the three PIPS sites based on similarity in prevalence of high-dose opioid patients, prevalence of sedative co-prescribing, and facility size. Outcomes for patients at control sites will be included in the models and designated by a control site indicator to help protect against confounding from secular trends and system-wide effects (e.g., policy changes). Coefficients estimated for the study period indicators will indicate the immediate changes in outcomes upon entering the PIPS facilitation period and the sustainment period, in comparison to the pre-facilitation period. The coefficients estimated for the time elapsed since the beginning of each study period will represent how PIPS facilitation impacted trends in outcomes over time in each of the three study periods.

We hypothesize significant increases in slopes in the facilitation period, compared to the pre-facilitation period, for the proportion of patients transitioning to safer opioid regimens and for the proportion of patients utilizing CIH treatments over time. We hypothesize that there will not be significant level changes in these outcomes at the onset of the PIPS facilitation period. We are unsure if there will be sufficient power to detect significant changes in slope or level in the maintenance period. If there is sufficient power, we would expect significant increases in outcome slopes in the maintenance period compared to the pre-facilitation period, but the magnitude of these changes would be smaller than those in the comparison of slopes in the facilitation vs. pre-facilitation periods. We hypothesize that significant level changes will not be present at the onset of the maintenance period compared to the facilitation period.

Our use of the ITS design and analytic strategy is in line with recommended best practices [33, 34]. The intervention will occur independently of other changes over time and is unlikely to affect data collection, as the data come from administrative data. The primary outcomes will be measured objectively (i.e., administrative data), covering 100% of the final sample at each time point. We prespecified the shape of the intervention effect and have described our sample calculations size in the power analysis section. We will be using time series regression models, accounting for and considering potential methodological issues associated with time series analyses (e.g., auto-correlation, time-varying confounders).

#### Power analysis

Based on the prevalence of eligible patients at the three sites, we expect approximately 150 patients to be eligible per site per month. We estimated the proportion of patients who change their opioid medication to a safer regiment without influence from PIPS to be 20% in the pre-facilitation period. Assuming a doubling of the patients moving to safer regimens during the pre-facilitation period to 40% within the PIPS facilitation and maintenance periods, setting alpha = .05, with a two-tailed test, we will have sufficient power (> 90%) to detect an odds ratio (without comparison sites) of 2.08 (95% CI 1.16, 3.75) with 130 patients per group (to allow for 20% attrition). There is no existing research from which to evaluate this effect size. However, given the expected wide range of readiness to adopt this clinical program, we anticipate this effect size is reasonable and clinically meaningful.

### Aim 3: Economic evaluation

A budget impact analysis will examine three categories of costs: (1) implementation, (2) PIPS clinical program, and (3) consequent costs. Implementation costs include staff, length of meeting time participation, and supplies. Clinical program costs include the time of the pharmacists who develop and manage dose tapering protocols, the letter mailing cost, and the extra time for the primary care provider to introduce PIPS and encourage other CIH treatments. Consequent cost represents the change in healthcare utilization costs from pre-PIPS to post-PIPS periods. Both implementation and clinical program costs will be measured through the micro-costing method [35], using an approach that is similar to a previous cost analysis of an implementation project in the VHA [36].

#### Study participants

Participants include patients described in the “Aim 2: Effectiveness evaluation” section as well as the pharmacists and primary care providers.

#### Data collection

Data will be obtained from the VHA’s administrative data and logs kept by study personnel. The logs will include information about the total staff FTE (full-time equivalent), length of meeting time, and number of participants by title of employment, relying on the VHA employment pay category and the average salary in each employment category to estimate cost. The logs will also include a list of supplies needed to implement PIPS, including equipment and materials (e.g., guides, instructions) as well as an “Other” category to document unexpected expenses.

#### Data analysis

To ascertain consequent cost, an ITS model like those described above for assessing PIPS clinical targets will be used to evaluate changes in healthcare utilization associated with the implementation of PIPS. Primarily, this design will compare post-PIPS (in the facilitation and sustainment periods) outcomes and trends to pre-PIPS levels within the three intervention sites; however, sensitivity analyses will be conducted to compare PIPS sites to closely matched control sites to guard against confusing PIPS effects with secular trends of disruptions affecting all sites in the health care system. We will account for varying lengths of the study periods of interest, separate types of healthcare utilization (pain management related care vs. care for other medical conditions), and regional variation in healthcare utilization in our model.

#### Project status

As of August 2018 (manuscript submission), PIPS is in the implementation phase, with two VA health care systems (sites) in the implementation phase since March 2017. A third VA health care system began the 18-month implementation phase in May 2018. The project team had difficulty identifying a third site, and the internal facilitator changed during the pre-implementation period at the third site. Pre-implementation formative evaluation was completed at all sites, and implementation/progress-focused formative evaluation was beginning at two sites. In both sites that began implementation in 2017, at least 91 patients have enrolled, with 3 patients enrolled at the site that began in 2018.

## Discussion

To address the growing opioid epidemic, it is important to understand optimal strategies for implementing evidence-based practices in primary care settings. As part of this hybrid, type III trial, we are evaluating the effectiveness of the implementation facilitation approach, which has been used previously to implement evidence-based practices in primary care [20]. The current trial will not only identify which activities within implementation facilitation are key to implementation of a collaborative care clinical program based in primary care (PIPS) but also evaluate the effectiveness of PIPS on key clinical outcomes. Quantitative and qualitative data collected as part of the stages of the formative evaluation will contribute to a more in-depth understanding of which factors are facilitating or limiting successful implementation. The ITS design will allow us to see how sites performed with respect to clinical outcomes before, during, and after the application of implementation facilitation. The findings from this study will inform spread of PIPS beyond the initial three health care systems, as well as the implementation of similar collaborative care, primary-care-based clinical programs.

In addition to enhancing the literature examining the effectiveness of implementation facilitation, including the integration of CFIR and implementation facilitation, the study also adds to fidelity measurement literature through the facilitation fidelity measurement.

## Limitations

There are some limitations that should be noted. First, the VHA has several initiatives that target opioid safety [11], including interventions focused on primary care, which will likely overlap with the study period. It is not possible to separate the impact of those initiatives from the impact of PIPS on clinical care. Moreover, clinical leadership and staff will have to respond to and continue to address VHA initiatives during the study period which may impact the ability of staff at our study locations to focus on PIPS implementation. Second, PIPS is being implemented in VHA and may not generalize to other healthcare systems, especially those that do not have clinical pharmacists integrated into primary care practice.

## Conclusion

There is growing recognition that the field of implementation science offers important guidance to increase the likelihood of success of care-delivery innovations such as PIPS, through identification of key factors influencing their adoption [37]. This study has the potential to (1) provide the field with an effective way in which to implement a collaborative care program designed to improve the safety and quality of pain treatment among veterans, (2) inform the spread of other collaborative care clinical programs, and (3) advance the use of implementation facilitation.

## Additional files

Additional file 1: Sample Vignette. (DOCX 12 kb)

Additional file 2: PIPS monthly facilitation log. (PDF 70 kb)

## Abbreviations

BIA: Budget impact analysis
CAVHS: Central Arkansas Veterans Healthcare System
CFIR: Consolidated Framework for Implementation Research
CIH: Complementary and integrative health
EBPPs: Evidence-based practices and programs
ECHCS: VA Eastern Colorado Health Care System
EF: External facilitation
FTE: Full-time equivalent
IF: Internal facilitation
ITS: Interrupted time series
MEDD: Morphine equivalent daily dose
ORIC: Organizational Readiness for Implementing Change
OSI: Opioid Safety Initiative
PIPS: Primary Care-Integrated Pain Support
RE-AIM: Reach, Effectiveness, Adoption, Implementation, and Maintenance
TVHS: Tennessee Valley Healthcare System
VHA: Veterans Health Administration
