# Supplementary material for: Testing implementation facilitation of a primary care-based collaborative care clinical program using a hybrid type III interrupted time series design: a study protocol
Source: Implement Sci. 2018 Nov 29;13:145. doi: 10.1186/s13012-018-0838-2 (PMC6262952; doi:10.1186/s13012-018-0838-2)
Supplement: Supplementary file 1 — Sample Vignette. (DOCX 12 kb) [file 13012_2018_838_MOESM1_ESM.docx]

Sample Vignette

Dr. Miller – External Facilitator

Dr. Jones – Internal Facilitator

Dr. Lee – Champion

On a project wide call early in the implementation phase, Dr. Jones mentions that Dr. Lee has been re-assigned to a leadership position at the VISN and will be leaving the medical center. Dr. Miller follows up with an ad hoc call with Dr. Jones to discuss next steps. *(Engages Internal Facilitator to support PIPS implementation)* On this call, Dr. Jones and Dr. Miller discuss who among the other PCPs at the site might be an appropriate person to take on the role of championing PIPS. *(Problem-solving/assessment of implementation barriers and facilitators)* They decide to approach Dr. Manuel, who enthusiastically agrees since she has had positive experiences with the program. *(Changing Implementation Team).* Having been an “end user” of the PIPS program, Dr. Manuel has noticed a couple things that could be improved. After discussing her suggestions with the team on an ad hoc call *(Problem-solving/assessment of implementation barriers and facilitators)*, she implements a few wording changes to the consult template to give PCPs a better opportunity to describe the situation. *(Changing record systems)* Dr. Manuel also notes that she was speaking with the new facility CMO at a “meet and greet” last week and that the CMO hadn’t heard of PIPS or other efforts in primary care to improve pain treatment. She prepares a brief presentation about the PIPS program and its potential benefits to patients and providers and presents it to the CMO and colleagues the following month. *(Academic detailing/education; Informing local opinion leaders)* She then places the presentation on the PIPS SharePoint so that teams at other sites can adapt and use it. *(Developing materials and adding them to a shared library)*
