# Supplementary material for: Testing implementation facilitation of a primary care-based collaborative care clinical program using a hybrid type III interrupted time series design: a study protocol
Source: Implement Sci. 2018 Nov 29;13:145. doi: 10.1186/s13012-018-0838-2 (PMC6262952; doi:10.1186/s13012-018-0838-2)
Supplement: Supplementary file 2 — PIPS monthly facilitation log. (PDF 70 kb) [file 13012_2018_838_MOESM2_ESM.pdf]

# PIPS monthly facilitation log

Please complete the survey below.

Thank you!

| | |
|---|---|
| Site location | ◯ Site 1<br>◯ Site 2<br>◯ Site 3<br>◯ Site 4 |
| Responder's initials | __________________ |
| Role of person taking survey | ◯ Champion<br>◯ Internal Facilitator<br>◯ External Facilitator |
| Month captured by log | ◯ January<br>◯ February<br>◯ March<br>◯ April<br>◯ May<br>◯ June<br>◯ July<br>◯ August<br>◯ September<br>◯ October<br>◯ November<br>◯ December |
| Year captured by log | __________________ |
| In the past month, did you participate in a project-wide team call? | ◯ Yes<br>◯ No |
| In the past month, did you participate in the Community of Practice call? | ◯ Yes<br>◯ No |
| In the past month, did you participate in a site-specific team call? | ◯ Yes<br>◯ No |
| In the past month, did you engage the external facilitator to support PIPS implementation? | ◯ Yes<br>◯ No |
| How many times during the past month did you engage the external facilitator to support PIPS implementation? | ◯ 1<br>◯ 2<br>◯ 3<br>◯ 4<br>◯ 5<br>◯ 6 or more |
| Please provide a brief description of your interactions with the external facilitator to support PIPS implementation during the past month. | ______________________ |
| Approximately how many minutes total did you spend engaging with the external facilitator to support PIPS during the past month? | __________________ |
| In the past month, did you engage the internal facilitator to support PIPS implementation? | ◯ Yes<br>◯ No |

How many times during the past month did you engage the internal facilitator to support PIPS implementation?

◯ 1
◯ 2
◯ 3
◯ 4
◯ 5
◯ 6 or more

Please provide a brief description of your interactions with the internal facilitator to support PIPS implementation during the past month.

__________________________________________

Approximately how many minutes total did you spend engaging with the internal facilitator to support PIPS during the past month?

__________________________________

In the past month did you engage the champion to support PIPS implementation?

◯ Yes
◯ No

How many times during the past month did you engage the champion to support PIPS implementation?

◯ 1
◯ 2
◯ 3
◯ 4
◯ 5
◯ 6 or more

Please provide a brief description of your interactions with the champion to support PIPS implementation during the past month.

__________________________________________

Approximately how many minutes total did you spend engaging with the champion to support PIPS during the past month?

__________________________________

In the past month, were academic detailing and/or education provided in connection with PIPS implementation at your site?

◯ Yes
◯ No

How many times during the past month were academic detailing and/or education provided at your site in connection with PIPS implementation?

◯ 1
◯ 2
◯ 3
◯ 4
◯ 5
◯ 6 or more

Please provide a brief description of the academic detailing and/or education provided at your site in connection with PIPS implementation during the past month.

__________________________________________

Approximately how many minutes total were spent providing academic detailing and/or education at your sight in connection with PIPS implementation during the past month?

__________________________________

In the past month, did you engage in problem-solving based on an assessment of implementation barriers and facilitators in connection with PIPS implementation?

◯ Yes
◯ No

How many times during the past month did you engage in problem solving based on an assessment of implementation barriers and facilitators?

◯ 1
◯ 2
◯ 3
◯ 4
◯ 5
◯ 6 or more

Please provide a brief description of PIPS related problem solving based on an assessment of implementation barriers and facilitators activities you engaged in during the past month.

__________________________________

Approximately how many minutes total did you spend engaging in problem solving based on an assessment of implementation barriers and facilitators in connection with PIPS implementation during the past month?

___________________________

In the past month, were audit and feedback provided at your site in connection with PIPS implementation?

◯ Yes
◯ No

How many times in the past month were audit and feedback provided in connection with PIPS implementation at your site?

◯ 1
◯ 2
◯ 3
◯ 4
◯ 5
◯ 6 or more

Please provide a brief description of the audit and feedback provided at your site in connection with PIPS implementation during the past month.

__________________________________

Approximately how many minutes total of audit and feedback were provided in connection with PIPS implementation during the past month?

___________________________

In the past month, did you capture and share local knowledge in connection with PIPS implementation with other sites?

◯ Yes
◯ No

How many times in the past month was local knowledge captured and shared at your site?

◯ 1
◯ 2
◯ 3
◯ 4
◯ 5
◯ 6 or more

Please provide a brief description of the local knowledge that was captured at your site and how it was shared during the past month.

__________________________________

Approximately how many minutes total did you spend capturing and sharing local knowledge in connection with PIPS implementation during the past month?

___________________________

In the past month were record systems relating to PIPS implementation changed?

◯ Yes
◯ No

How many times in the past month were record systems relating to PIPS implementation changed?

◯ 1
◯ 2
◯ 3
◯ 4
◯ 5
◯ 6 or more

Please provide a brief description of changes made to PIPS-related record systems at your site during the past month. __________________________________

Approximately how many minutes did you spend changing PIPS related record systems during the past month? __________________________

In the past month were PIPS marketing materials distributed at your site?
- ◯ Yes
- ◯ No

How many times in the past month were PIPS marketing materials distributed at your site?
- ◯ 1
- ◯ 2
- ◯ 3
- ◯ 4
- ◯ 5
- ◯ 6 or more

Please provide a brief description of the PIPS marketing materials and how they were distributed at your site during the past month. __________________________________

Approximately how many minutes did you spend distributing PIPS marketing materials at your site during the past month? __________________________

In the past month did the PIPS clinical team change at your site?
- ◯ Yes
- ◯ No

How many times in the past month did the PIPS clinical team change at your site?
- ◯ 1
- ◯ 2
- ◯ 3
- ◯ 4
- ◯ 5
- ◯ 6 or more

Please provide a brief description of PIPS clinical team changes at your site during the past month. __________________________________

Approximately how many minutes did you spend in connection with PIPS clinical team changes during the past month? __________________________

In the past month, did the PIPS implementation team change at your site?
- ◯ Yes
- ◯ No

How many times in the past month did the PIPS implementation team change at your site?
- ◯ 1
- ◯ 2
- ◯ 3
- ◯ 4
- ◯ 5
- ◯ 6 or more

Please provide a brief description of PIPS implementation team changes at your site during the past month. __________________________________

Approximately how many minutes did you spend in connection with PIPS implementation team changes at your site during the past month? __________________________

In the past month, did you develop PIPS materials relating to PIPS implementation and add them to the SharePoint folder?
- ◯ Yes
- ◯ No

How many PIPS materials were developed at your site and added to the SharePoint folder during the past month?

- ◯ 1
- ◯ 2
- ◯ 3
- ◯ 4
- ◯ 5
- ◯ 6 or more

Please provide a brief description of the PIPS materials that you developed and added to the SharePoint folder during the past month.

__________________________________

Approximately how many minutes did you spend developing PIPS materials and adding them to the SharePoint during the past month?

__________________________________

In the past month did you provide information about PIPS to local opinion leaders?

- ◯ Yes
- ◯ No

How many times in the past month did you provide PIPS information to local opinion leaders?

- ◯ 1
- ◯ 2
- ◯ 3
- ◯ 4
- ◯ 5
- ◯ 6 or more

Please provide a brief description of the PIPS information that you provided to local opinion leaders during the past month and the circumstances under which it happened.

__________________________________

Approximately how many minutes did you spend providing information about PIPS to local opinion leaders during the past month?

__________________________________

In the past month, did you engage national and/or regional policy makers to support PIPS implementation?

- ◯ Yes
- ◯ No

How many times in the past month did you engage national and/or regional policy makers to support PIPS implementation?

- ◯ 1
- ◯ 2
- ◯ 3
- ◯ 4
- ◯ 5
- ◯ 6 or more

Please provide a brief description of the ways in which you engaged national and/or regional policy makers in support of PIPS implementation during the past month.

__________________________________

Approximately how many minutes did you spend engaging national and/or regional policy makers in support of PIPS during the past month?

__________________________________

In the past month, did you provide technical assistance and/or coaching to promote high quality implementation of PIPS at your site?

- ◯ Yes
- ◯ No

How many times during the past month did you provide technical assistance and/or coaching to promote high quality implementation of PIPS at your site?

- ◯ 1
- ◯ 2
- ◯ 3
- ◯ 4
- ◯ 5
- ◯ 6 or more

Please provide a brief description of the technical assistance and/or coaching that you provided in connection with PIPS implementation at your site during the past month. __________________________________________

Approximately how many minutes did you spend providing technical assistance and/or coaching to promote high quality implementation of PIPS at your site during the past month? __________________________________
